# Competitive Abilities in Experimental Microcosms Are Accurately Predicted by a Demographic Index for R*

**DOI:** 10.1371/journal.pone.0043458

**Published:** 2012-09-06

**Authors:** Ebony G. Murrell, Steven A. Juliano

**Affiliations:** 1 Department of Entomology, University of Wisconsin-Madison, Madison, Wisconsin, United States of America; 2 School of Biological Sciences, Illinois State University, Normal, Illinois, United States of America; Institut Pasteur, France

## Abstract

Resource competition theory predicts that R*, the equilibrium resource amount yielding zero growth of a consumer population, should predict species' competitive abilities for that resource. This concept has been supported for unicellular organisms, but has not been well-tested for metazoans, probably due to the difficulty of raising experimental populations to equilibrium and measuring population growth rates for species with long or complex life cycles. We developed an index (R_index_) of R* based on demography of one insect cohort, growing from egg to adult in a non-equilibrium setting, and tested whether R_index_ yielded accurate predictions of competitive abilities using mosquitoes as a model system. We estimated finite rate of increase (λ′) from demographic data for cohorts of three mosquito species raised with different detritus amounts, and estimated each species' R_index_ using nonlinear regressions of λ′ vs. initial detritus amount. All three species' R_index_ differed significantly, and accurately predicted competitive hierarchy of the species determined in simultaneous pairwise competition experiments. Our R_index_ could provide estimates and rigorous statistical comparisons of competitive ability for organisms for which typical chemostat methods and equilibrium population conditions are impractical.

## Introduction

**Figure 1:**
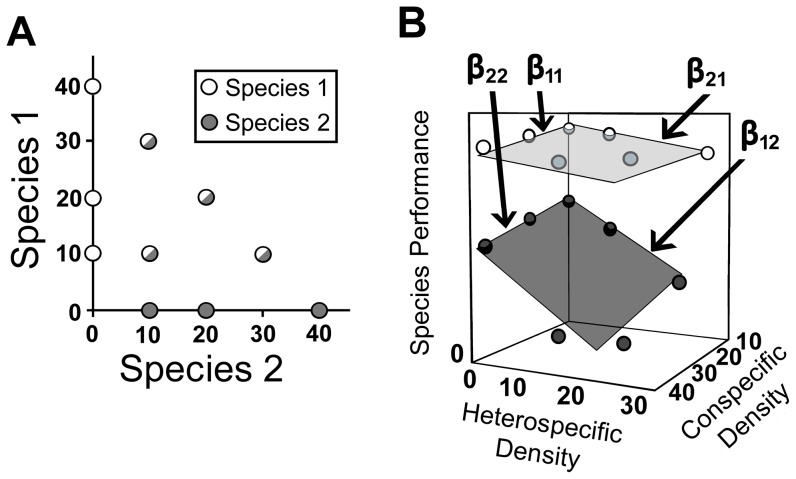
Diagram of a response surface design experiment between 2 species (no actual data shown). (A) Multiple cohorts of intraspecific (solid circles) and interspecific combinations (bicolored circles) of species are established, with a standard amount of resource provided to each cohort. (B) The linear responses (â) of each species' performance to both conspecific and heterospecific densities are estimated via multiple regressions. The dots represent data obtained from each cohort shown in A, while the planes represent the slope estimates from the linear regressions.

Interspecific resource competition is thought to be a major driving force of community composition [1], [2]. Relative competitive abilities of species have been assessed using a variety of methods, such as substitutive designs, additive designs, and response surface designs [3]. Of these designs, the response surface design is particularly noteworthy due to its thoroughness. Under this experimental design, two or more species are reared in multiple treatments, each treatment containing a cohort of a single species or multiple species at standard ratios and densities (**Fig. 1A**). The competitive response of each species to conspecific and heterospecific densities is then assessed among treatments. If sufficient treatments are used, it is possible to estimate the quantitative relationships of each species' performance to increasing densities via regressions (**Fig. 1B**), and to then determine competitive ranking of the species based upon the relative inter- and intraspecific competitive effects of each species [4]. The response surface design is thorough and particularly useful when the nature of the competitive interactions (interference, resource competition, etc.) is unknown. Despite or perhaps because of its thoroughness, the response surface design is used on a limited number of systems [5], [6], [7], as it requires many organisms and replicates to assess competition for even 2–3 species. Assessing relative competitive abilities among multiple species with this method would be logistically prohibitive, and therefore it is impractical to use to assess competitive relationships in a diverse community.

In cases where resource competition occurs, resource competition theory (RCT [1], [8], [9]) yields a more elegant predictor of competitive ability: R*. Assuming that a population of organisms has a resource-independent mortality rate (*m*), R* is the equilibrium level of a single limiting resource – or single resource environment [10] – at which population growth dN/dt = 0 and resource-dependent production balances *m*. RCT predicts that for any group of species competing for a single limiting resource, the species with the lowest R* should be the best competitor, as that species can maintain an equilibrium population at lower resource levels than any other species [1], [9].

RCT and R* have proven to be powerful predictive tools for understanding competitive ability for microorganisms, algae, and zooplankton in chemostats or other artificial systems in which populations can be maintained until equilibrium is reached (e.g., [1], [9], [11], [12], [13], [14]), and to a lesser extent in terrestrial herbaceous plant systems [10], but rarely for organisms with complex life cycles. This body of theory is presumed to be generally valid for nearly all organisms [2], and could provide insight into the structure of many communities of resource competitors. Why, then, is the successful estimation and application of R* in non-microbial communities so rare [15]?

The most likely answer is that for many species, population growth rates and equilibrium values like R* are difficult to measure. Species that are large, long-lived, or have complex life cycles are difficult to raise exclusively in a highly regulated environment such as a chemostat, and raising successive generations of such species to population equilibrium may be impractical for most researchers. Competition among such species is more commonly investigated in nonequilibrium microcosms, or “bottle” experiments [16], using methods such as the response surface design.

If R* is to be generally applied as a predictor of competitive ability, an alternative method is clearly needed to estimate R* for a wider array of organisms. One such alternative method was developed by Tilman and Wedin [10] for estimating R* and competitive abilities for plants growing in old-fields. “Food thresholds” have also been estimated from calculated growth rates of cladoceran species [17] and such thresholds likely to be useful indices of R*. However, to date there is no method for approximating R* that is suitable for animals with complex life cycles (e.g., insects, amphibians). Further, no one has determined whether indices of R* accurately predict competitive rankings obtained from other experimental designs for estimating competitive ability.

We propose, as an alternative to measuring resource level at population equilibrium, a method that uses demographic data from a single cohort to estimate population growth under a range of resource environments under nonequilibrium conditions. If cohorts are raised at different levels of resource availability, and demographic data are used to estimate population growth for each resource level, regression can be used to describe the relationship of estimated population growth rate to resource amount, and to create an index of the resource abundance necessary for a stable population. Values of that index for multiple species can then be used to generate testable predictions of competitive outcomes as outlined by Tilman [1]. Such indices, if possible to obtain, would be extremely useful for testing competitive ability in a larger variety of organisms, and among larger arrays of species, than can practically be done using current methods.

We tested this new method for creating an index of R* with three species of mosquitoes that compete as aquatic larvae: *Aedes albopictus* (Skuse), *Aedes aegypti* (L.), and *Culex pipiens* (L.). Mosquitoes are a good model system for this research because: (A) Many species, including the ones we used, are known resource competitors (reviewed by [18], [5]), (B) They are easily raised from egg to adult in laboratory microcosms, with controlled detritus resource amounts and environmental conditions, (C) Methods for testing competitive ability among species are well-established (reviewed by [5]), (D) Methods for estimating population growth from demographic data are commonly used [19], [20], [21], [22], [23]. Among the three species we use, *A. albopictus* has been generally shown to be a superior competitor to *A. aegypti* and *C. pipiens* (reviewed by [18], [5]). Very limited data suggest that *A. aegypti* is also a superior competitor to *C. pipiens* (reviewed by [5]).

Most mosquito larvae are filter feeders and browsers on fine particulate organic matter (FPOM), including microorganisms and fine detritus [24]. Among our three study species, *A. aegypti* and *A. albopictus* typically spend more time browsing at the bottom of the container, and *Culex* spend more time at the surface [24]; however, all three species adjust their browsing and filter-feeding behavior as necessary to acquire resources [25], [26]. These species also gnaw on dead animal matter (e.g., insect carcasses) if it is sufficiently soft, but the majority of their nutrition is typically obtained from the microorganisms and FPOM in the water column [27].

Typical experiments with populations of mosquito larvae manipulate resource availability by manipulating abundance of plant and animal detritus, and thereby manipulating availability of microorganisms and FPOM (reviewed by [5]). This experimental approach means that typical experiments actually involve three trophic levels (detritus, microorganisms, mosquitoes). Though the manipulation of detritus rather than direct manipulation of resource may seem to be an unnecessary complication, detritus decomposes at a consistent rate under laboratory conditions, and this decay rate directly correlates with microbial growth [22]. Thus, the detritus provides a resource base for the mosquitoes over an extended period of time, but this resource is still finite because of the limitation of the amount of detritus added. Furthermore, studies not only in mosquito systems [28], [29], but also in detritus-based terrestrial systems [30], [31] and aquatic cave communities [32] have shown a bottom-up increase in FPOM consumer numbers and consumer performance when either the amount or nutritional quality of the detritus is increased. Based upon these studies, we infer that manipulation of the detritus is a reasonable alternative to direct resource manipulation for FPOM consumers in detritus-based systems, and can therefore be used to assess resource competitive ability in mosquitoes.

In this study, we test the prediction that our demographic index of R* (R_index_) can predict competitive abilities among mosquito species. We describe two types of experiments, one to quantify R_index_ for three mosquito species, and one to determine competitive advantage for pairs of species in the chosen resource environment. Conducting both types of experiments concurrently allows us to test whether R_index_ predicts abilities of resource competition as accurately as the already-accepted method of response-surface design experiments.

## Methods

### Origins and maintenance of mosquitoes used

This experiment was conducted in two temporal blocks lasting 54 and 53 days each. For both blocks, all 3 species used were obtained as eggs from colonies reared in our lab; none of these colonies were more than 4 generations removed from the field. *Aedes albopictus* and *A. aegypti* (1^st^ lab generation from colonies originating in Tampa, Florida, USA) eggs were hatched synchronously from stored egg papers using 0.4 g/L hatching medium [22]. Because *Culex pipiens* eggs cannot be stored, it is more difficult to collect and to hatch *C. pipiens* eggs synchronously [33]. To gather as many eggs as possible, we withheld oviposition cups from our *C. pipiens* colonies (first block: unknown generation, origin Springfield, IL USA and East St. Louis, IL USA, collected summer 2007; second block: unknown generation, Springfield, IL USA, collected summer 2008) while bloodfeeding the colony intensively for 1 week. Three days prior to the experiment, we placed oviposition cups inside the colony to encourage simultaneous oviposition of multiple egg rafts [33]. All egg rafts collected 2 days prior to the experiment were then placed in 0.4 g/L hatching medium.

Once hatched, all larvae were added to each replicate microcosm as 1^st^ instars. Replicate microcosms consisted of 250-mL plastic beakers filled with 200 mL nanopure water, 500 µL of water obtained from natural tree holes (to standardize initial bacteria inoculum), and detritus, which consisted of 95% by mass senescent white oak (*Quercus alba*) leaves and 5% dead nymphal crickets (*Gryllodes sigillatus*). All detritus was dried >24 hours at 50°C; leaf detritus was broken into pieces approximately 2–5 mm^2^ and mixed prior to weighing. Containers were incubated with detritus, water, and inoculum for 3 days prior to addition of larvae.

All replicates for each block were housed in a single environmental chamber at 25°C (±2°C), 14∶10 L∶D cycle. Starting on day 5, containers were checked daily for pupae, which were isolated prior to eclosion. For each individual adult we recorded species, sex, container of origin, and number of days to eclosion. For each female, we recorded dry mass and wing length.

We then calculated estimated instantaneous rate of increase for each container using Livdahl and Sugihara's [19] index of performance (r′):
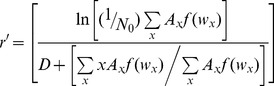
(1)The numerator of this equation estimates the net reproductive rate of the cohort, whereas the denominator estimates the mean cohort generation time [19], [34]. This equation yield accurate estimates of per capita rate of increase [34]. *N_0_* is the initial number of females (assumed to be 50% of the larvae), *A_x_* is the number of females eclosing on day *x*, *D* is the estimated number of days from eclosion to adulthood and oviposition, and *f*(*w_x_*) is the predicted fecundity of females of mean wing length eclosing on day *x* (*w_x_*). Female mosquito wing length is an accurate predictor of fecundity within species ([35], [23], [36], **Table 1**). We used published regressions to generate *f*(*w_x_*) for females of each species on each day *x* (**Table 1**). We then estimated for each container the finite rate of increase from r′ as λ′ = exp(r′). Using λ′ enables us to estimate population rate of increase from containers with no surviving females (λ′ = 0), which would be inestimable using r′ (r′ = −∞) [21].

**Table 1:** Wing length-fecundity functions *f(w_x_)* and *D* values for the three mosquito species, and the studies from which these values and functions were derived.

Species	Function	D	Data/function Source
*A. aegypti*	*f(w_x_)* = 0.5*(2.50*w^3^−8.616)	12	Briegel 1990 [47]
*A. albopictus*	*f(w_x_)* = 0.5*(78.02*w−121.240)	14	Lounibos et al. 2002 [48]
*C. pipiens*	*f(w_x_)* = 0.5*(46.83*w−104)	4.5	Vinogradova and Karpova 2006 [49]

In all cases w = wing length in mm.

This estimate of λ′ depends primarily on survivorship of females, and less dependent on variation in fecundity-size slopes [21]. Leisnham et al. [23] found that there is variation in *f*(*w_x_*) among populations of *A. albopictus*; however, the competitive abilities predicted by Leisnham et al. [23] were insensitive to whether separate *f*(*w_x_*) for each population were used vs. a single pooled *f*(*w_x_*) for all populations. Leisnham and Juliano [36] also determined that *f*(*w_x_*) for *A. aegypti* did not vary significantly among eight different populations.

### R_index_ Experiment

Each species was raised in initial single species densities of 40 larvae/container. Each experimental microcosm held 0.5 g, 1.0 g, or 1.5 g detritus, for a total of 9 treatments. This experiment was run concurrently with the competition experiment (below) in 2 blocks, with 2–4 replicates of each treatment per block. Variation in the number of replicates was due to the availability of *C. pipiens*

For each species we used nonlinear least squares (PROC NLIN, SAS 9.1) to estimate the functional relationship between λ′ and detritus amount. The hyperbolic relationship derived from mechanistic models [1] did not yield a good fit to our data. We used instead a phenomenological polynomial model, starting with a quadratic function, and testing whether polynomials of increasing order yielded better fit. We found a quadratic function provided the most parsimonious fit for our data. As our goal is to estimate R_index_ with confidence limits, the form of the function is not critical. We simply need an estimate of the value of resource amount (Detritus) at which the curve crosses the zero-growth value of λ′ = 1. We used the following equation, which provides such an estimate in place of the standard regression estimate of the *y* intercept:
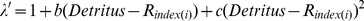
(2)where the independent variable Detritus is initial detritus amount, and model parameters are R_index(i)_ = detritus amount for species *i* at which predicted λ′ = 1, and *b* and *c*, which are phenomenological parameters of the polynomial estimated by PROC NLIN. This form, used with NLIN has the desirable property of yielding direct estimates of R_index(i)_, with confidence intervals, and facilitates statistical comparison of R_index(i)_ among species.

We tested the differences between pairs of species in R_index_ using PROC NLIN with an extension of eq. **(2)** as an indicator variable model [37] for each pair of species. This indicator variable model was:
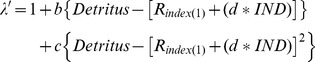
(3)where *d* is the difference between R_index_ values for the two species (R_index(2)_ = R_index(1)_+*d*) and IND is an indicator variable (i.e., IND = 0 for Species 1, IND = 1 for Species 2). Lower and upper 95% confidence intervals (CIs) on *d* that did not include 0 were used as a statistical test for a significant pairwise difference between R_index_ values. The results of these pairwise tests were used to determine the competitive ranking of species by their R_index_ values.

### Competition Experiment

Initial detritus amount for each replicate microcosm was 0.5 g. Treatments included low, medium, and high single species densities (10, 20, and 40 larvae respectively) for each species, and four different two-species combinations for each possible pairwise combination of competitors (10∶10, 20∶20, 10∶30, and 30∶10 larvae). There were 2–3 replicates per block. Variation in replicate numbers was due to the availability of *C. pipiens* (see previous section).

We did not test three-way combinations of species, as we were interested in only the competitive rank of each species to every other species individually. Since each species' R_Index_ was compared to every other species' R_Index_ using a pairwise analysis, pairwise competition experiments provided the best direct comparison with the R_Index_ results. We used a general linear model (PROC GLM, SAS 9.1) to analyze the response of each species' λ′ to the inter- and intraspecific densities (continuous variables), block, and all block*density interactions. The raw data fit the assumptions of equal variance and normality. The direction and significance of intraspecific and interspecific competitive effects of each species were assessed based on slope parameters relating λ′ to densities of conspecifics or heterospecifics (i.e., significant negative slopes indicate a significant effect of competition on λ′). Comparison of inter- and intraspecific competitive effects is the key to determining which species (if any) may have a competitive advantage, or if stable coexistence is possible [38], [4]. Though the general linear model analysis produces estimates of these effects (i.e., the slopes relating λ′ to conspecific or heterospecific density), and tests whether those effects differ from 0, such analyses are not ideal for comparing intra- and interspecific competitive effects, because those effects are estimated in different ANOVAs (i.e., the interspecific effect of a species comes from analysis of λ′ of the other species, whereas the intraspecific effect of a species comes from analysis of λ′ of that species) [4]. We compared the magnitudes of intra- and interspecific effects by tabulating estimates from multiple analyses and informally comparing estimates and confidence intervals. Strong competitive advantage is indicated when one competitor's interspecific effect is much greater (e.g., more negative) than its intraspecific effect, and the other competitor's interspecific effect is much less than its intraspecific effect [38], [4]. Using this criterion, we ranked the species for competitive ability based on competitive effect estimates. These rankings were then qualitatively compared to the estimates from the R*_index_ experiment.

## Results

### R_index_ Experiment

**Figure 2:**
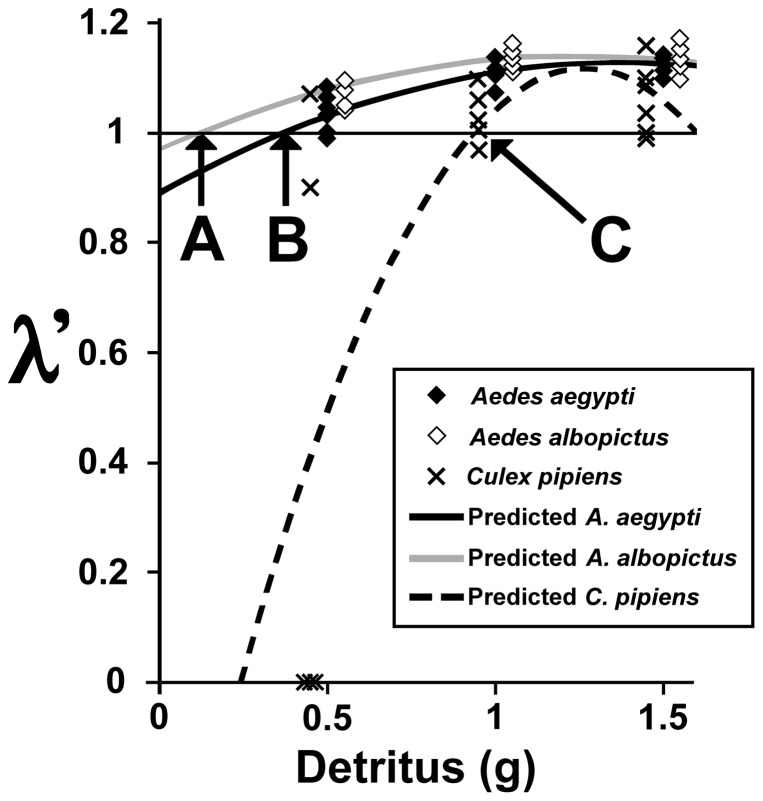
Experimental response of mean λ′ for each species by detritus amounts. Data points for *A. albopictus* and *C. pipiens* are offset. The curves represent the quadratic function for each species, while the arrows indicate values of *x* at which λ′ = 1.0 (R_Index_) for *A. albopictus* (**A**), *A. aegypti* (**B**), and *C. pipiens* (**C**).

Values of R_index_ (**Table 2**) differed significantly among the three species (**Table 3**). *Aedes albopictus* had the lowest R_index_, followed by *A. aegypti* and *C. pipiens* (**Table 2** **,** **Fig. 2**). Relative resource competitive ability of the three species, based on these results, is thus predicted to be *A. albopictus*>*A. aegypti*≫*C. pipiens*.

**Table 2:** Parameter estimates of Detritus at λ′ = 1 (R_Index_, in dry g litter) for the three mosquito species, as well as estimates of the linear component (*b*) and the quadratic component (*c*) of each equation.

Parameter	Estimate	Standard Error	Approximate 95% Confidence Limits	
			Lower	Upper
*A. aegypti p<0.0001*				
**Detritus at λ′ = 1 (R_Index_)**	**0.3735**	**0.0588**	**0.2500**	**0.4971**
**B**	**0.2510**	**0.0492**	**0.1477**	**0.3543**
**C**	**−0.1248**	**0.0472**	**−0.2240**	**−0.0255**
*A. albopictus p = 0.0001*				
Detritus at λ′ = 1 (R_Index_)	0.1243	0.1081	−0.1029	0.3514
**B**	**0.2451**	**0.0491**	**0.1419**	**0.3482**
**C**	**−0.1085**	**0.0400**	**−0.1926**	**−0.0244**
*C. pipiens p = 0.0144*				
**Detritus at λ′ = 1 (R_Index_)**	**0.9355**	**0.1603**	**0.5893**	**1.2817**
b	0.7050	0.4272	−0.2179	1.6279
c	−1.0587	0.5825	−2.3171	0.1997

Significant parameter estimates/differences are in **bold print**.

**Table 3:** Estimates of the differences between Detritus at λ′ = 1 (R_Index_, in dry g litter) for the three mosquito species.

Comparison	Estimate of differences in R_Index_	Standard Error	Approximate 95% Confidence Limits	
			Lower	Upper
*A. albopictus* vs. *A. aegypti*	0.2689	0.0779	0.1112	0.4265
*A. albopictus* vs. *C. pipiens*	0.7155	0.2263	0.2552	1.1758
*A. aegypti* vs. *C. pipiens*	0.6002	0.1944	0.2048	0.9957

All three comparisons were significantly different from zero.

### Competition Experiment

Competitive asymmetry was evident for all pairs of mosquito species. For *A. albopictus*, λ′ showed a significant Block*A. aegypti interaction (**Table 4A**). There was a negative response to *A. aegypti* density in the linear model only in one block, and in the other block, *A. albopictus* was unaffected by *A. aegypti* (**Table 5**). Additionally, *A. albopictus* was not significantly affected by conspecific densities or *C. pipiens* density. For *A. aegypti*, λ′ showed a significant block*A. albopictus interaction (p = 0.0226, **Table 4B**). The effect of *A. albopictus* on *A. aegypti* was negative in both blocks, but much more so in Block 2 than in Block 1. Effects of intraspecific and *C. pipiens* densities were not significant (**Table 4B**).

**Table 4:** Linear model results and parameter estimates for effects of block and species densities on λ′ for (A) *Aedes albopictus*, R^2^ = 0.2837, (B) *Aedes aegypti*, R^2^ = 0.4979 (C) *Culex pipiens*, R^2^ = 0.3012.

A			
Source	DF	F Value	Pr>F
**Block**	**1**	**5.63**	**0.0211**
*A. albopictus*	1	1.56	0.2164
***A. aegypti***	**1**	**6.78**	**0.0118**
*C. pipiens*	1	0.78	0.3815
Block*A. albopictus	1	3.77	0.0573
Block* ***A. aegypti***	**1**	**7.35**	**0.0089**
Block*C. pipiens	1	2.75	0.1028
Error	55		

B			
Source	DF	F Value	Pr>F
Block	1	0.02	0.8982
***A. albopictus***	**1**	**7.93**	**0.0067**
*A. aegypti*	1	0.52	0.4748
*C. pipiens*	1	0.01	0.9197
Block* ***A. albopictus***	**1**	**5.15**	**0.0271**
Block*A. aegypti	1	0.12	0.7354
Block*C. pipiens	1	0.20	0.6557
Error	57		

C			
Source	DF	F Value	Pr>F
Block	1	0.15	0.7009
***A. albopictus***	**1**	**8.59**	**0.0050**
***A. aegypti***	**1**	**6.87**	**0.0114**
***C. pipiens***	**1**	**8.37**	**0.0055**
Block*A. albopictus	1	0.17	0.6853
Block*A. aegypti	1	0.36	0.5533
Block*C. pipiens	1	1.51	0.2244
Error	57		

Significant effects are in **bold face**.

**Table 5:** Estimates of intra- and interspecific competitive effects for the pairwise response surface experiments *Aedes albopictus* vs. *Aedes aegypti*.

Source	β_aa_ (Intraspecific)	SE	β_ae_ (Interspecific)	SE
*A. albopictus* Block 1	−0.00106	0.00047	−0.00103	0.00265
*A. albopictus* Block 2	0.00023	0.00047	**−0.00953**	0.00265
*A. albopictus* Combined	−0.00040	0.00035	**−0.00527**	0.00193

Source	β_ee_ (Intraspecific)	SE	β_ea_ (Interspecific)	SE
*A. aegypti* Block 1	−0.00183	0.00247	−0.00191	0.00051
*A. aegypti* Block 2	**−0.00066**	0.00242	0.00004	0.00051
*A. aegypti* Combined	**−0.00121**	0.00178	−0.00094	0.00038

For β subscripts the first subscript indicates the species having a competitive effect and the second subscript indicates the species being affected: *a* = *A. albopictus*, *e = A. aegypti*. **Bold face** indicates whether the inter- or intraspecific effect is greater (i.e., more negative) within a row.

Comparing the slope estimates that show the inter- and intraspecific effects of *A. albopictus* (**Table 5**) and *A. aegypti* (**Table 6**) shows that in Block 1, the two species are virtually identical in competitive effects. In contrast, in Block 2 *A. albopictus* had interspecific effect>>than intraspecific effect, whereas *A. aegypti* had interspecific effect≪than intraspecific effect (by more than an order of magnitude in both cases; **Table 5** **,** **6**). Combined effects from both blocks also show *A. albopictus* should be the superior competitor to *A. aegypti* (**Table 5** **,** **6**). We conclude from these data that *A. albopictus* is generally a superior resource competitor to *A. aegypti*; however, the two species should be relatively close to one another in competitive ability.

**Table 6:** Estimates of intra- and interspecific competitive effects for the pairwise response surface experiment *Aedes albopictus* vs. *Culex pipiens*.

Source	β_aa_ (Intraspecific)	SE	β_ap_ (Interspecific)	SE
*A. albopictus* Block 1	−0.00106	0.00047	**−0.02485**	0.01055
*A. albopictus* Block 2	0.00023	0.00047	**−0.01878**	0.01050
*A. albopictus* Combined	−0.00040	0.00035	**−0.02190**	0.00744

Source	β_pp_ (Intraspecific)	SE	β_pa_ (Interspecific)	SE
*C. pipiens* Block 1	**−0.01161**	0.00970	−0.00030	0.00051
*C. pipiens* Block 2	**−0.02934**	0.01014	0.00098	0.00057
*C. pipiens* Combined	**−0.01924**	0.00695	0.00033	0.00040

For β subscripts the first subscript indicates the species having a competitive effect and the second subscript indicates the species being affected: *a* = *A. albopictus*, *p = Culex pipiens.* **Bold face** indicates whether the inter- or intraspecific effect is greater (i.e., more negative) within a row.

For *C. pipiens*, λ′ showed a significant negative response to conspecific, *A. albopictus*, and *A. aegypti* densities (p = 0.0059) (**Table 4C**). Magnitudes of the significant negative effects of densities on *C. pipiens* were much greater than on either *A. albopictus* or *A. aegypti* (**Table 4C**). Interspecific competitive effects of *C. pipiens* were always less (by an order of magnitude or more) than its intraspecific competitive effects, regardless of which *Aedes* species was considered (**Table 7**). Further, interspecific effects of either *Aedes* species on *C. pipiens* were always much greater (again, by an order of magnitude or more) than their corresponding intraspecific effects. These competition experiments thus indicate that *C. pipiens* highly inferior as a competitor to either *Aedes*. Ranking of species by competitive ability are therefore: *A. albopictus*>*A. aegypti*>>*C. pipiens*.

**Table 7:** Estimates of intra- and interspecific competitive effects for the pairwise response surface experiment *Aedes aegypti* vs. *Culex pipiens*.

Source	β_ee_ (Intraspecific)	SE	β_ep_ (Interspecific)	SE
*A. aegypti* Block 1	−0.00183	0.00247	**−0.02359**	0.01056
*A. aegypti* Block 2	−0.00066	0.00242	**−0.01483**	0.01018
*A. aegypti* Combined	−0.00121	0.00178	**−0.01947**	0.00732

Source	β_pp_ (Intraspecific)	SE	β_pe_ (Interspecific)	SE
*C. pipiens* Block 1	**−0.01161**	0.00970	0.00065	0.00047
*C. pipiens* Block 2	**−0.02877**	0.01007	0.00008	0.00047
*C. pipiens* Combined	**−0.01924**	0.00695	0.00017	0.00194

For β subscripts the first subscript indicates the species having a competitive effect and the second subscript indicates the species being affected: *e = A. aegypti, p = Culex pipiens*. **Bold face** indicates whether the inter- or intraspecific effect is greater (i.e., more negative) within a row.

## Discussion

In our microcosm studies, relative competitive ranking of *A. albopictus*>*A. aegypti*>>*C. pipiens*, as predicted by R_index_, match exactly the competitive ranking produced in our pairwise competition experiments. Further, these rankings are consistent with previous competition experiments on these species (e.g., [21], [37], [22], [5]) that show that both *Aedes* are superior competitors to *Culex*, and that *A. albopictus* and *A. aegypti* are similar in competitive ability, with *A. albopictus* usually having an advantage. The disparity in competitive abilities for *Aedes* vs. *Culex* could be interpreted as, in part, a result of inter-generic differences in foraging patterns, with *Culex* more strictly a filter feeder and spending more time at the surface, whereas these *Aedes* feed by filtering and browsing often below the surface [24], [25], [26]. Thus we might expect the impact of resource competition from *Culex* on *Aedes* to be relatively small, as *Aedes* have access to a resource (browsable microorganisms) that is little used by *Culex*. Our data clearly support our prediction that our demographic index of R*, which we have called R_index_, provides a good prediction of competitive abilities.

Our method for estimating R* is novel in several ways. First, we were able to use demographic data from microcosm experiments to estimate R* from a single cohort of animals with a complex life cycle, rather than maintaining populations over multiple generations and estimating R* from equilibrium conditions. Our method could prove valuable for estimating resource competitive ability and for testing resource competition theory in species that can be raised in microcosms for one generation, but for which maintaining populations over multiple generations may be prohibitive or impossible. Our approach is not dependent upon a specific method for estimating population rate of increase (λ′ in our case). The index of performance we used (**eq. 1**) is often used for mosquitoes, but other ways of estimating rate of increase could be substituted. Taxa for which demographic estimates of population rate of increase have already been described include not only mosquitoes [19], [21], but also mayflies and damselflies [38], and species with competition at multiple life stages, such as annelids [39], [40]. For additional species it will be necessary to develop appropriate demographic estimates of population rate of increase suitable for single generations in microcosms. Such estimates in general require some knowledge of survivorship and fecundity of survivors, simplifying assumptions about other life history events and tradeoffs (e.g., that adult longevity, which we did not measure, is unaffected by the larval rearing environment), and ability to apply basic life table methods [19], [38], [34]. Species to which our method may be applied include most insects, anurans, short-lived invertebrates (e.g., spiders, freshwater crustaceans), annual plants, and possibly some fish – any species for which population growth rate can be estimated using life table methods over a single generation within experimental microcosms.

Second, our decision to use λ′ rather than r′ as an estimate of growth was useful for comparing species that vary greatly in their competitive response. Had we used r′ we would have been forced to omit replicates with no surviving females (λ′ = 0), which were particularly common in cases of extreme competitive asymmetry, such as *A. albopictus* and *C. pipiens* (14/22 replicates in the pairwise competition experiments yielded no surviving *C. pipiens* females). Failure to produce females was also common for *C. pipiens* at the lowest detritus resources level (**Fig. 2**). These values are meaningful because failure to produce surviving females indicates a severe impact of interspecific competition or low food. Though for our purposes, using the estimated finite rate of increase λ′ was preferable, our general demographic approach to an index of R* could be implemented using either λ′ or r′, or indeed other demographic estimates of rate of increase (e.g., [38]).

Third, our index of R* relied upon manipulations of detritus amount, rather than the food resource itself (i.e., the consumer-prey microorganisms). Manipulating detritus not only enables us to compare our results with previous microcosm experiments on competitive asymmetries (reviewed by [5]), it also simulates natural conditions. In natural water-filled containers, inputs of detritus provide the nutrients that fuel microbial growth [41], [42], [43] and natural variation in detritus inputs is related to on both variation in microbial populations and variation in mosquito population performance [44], [41]. Additionally, this approach could be used to test other predictions of resource-ratio theory [15] for detritus-based systems. For example, ratios of detritus types could be manipulated to test whether competitive abilities, and presumably indices of R*, vary accordingly. Manipulations of detritus ratios [45] and detritus quality [22] are known to affect mosquito competition, but effects of natural variation in detritus inputs have never been investigated in the context of resource competition theory and R* (but see [45]).

Although our microcosm experiments rely upon manipulation of detritus, our calculation of R_index_ based on a single cohort could be easily applied to systems where the resource is manipulated directly. The methods we have described are not specific to assessing competitive ability in detritus-based microcosm experiments; they could also be used to determine R_index_ for any organism for which demographic data from a single cohort can be used to estimate rate of increase, and for which resource abundance can be directly experimentally manipulated. Plants reared in greenhouse or growth chamber experiments, or in manipulated field plots, or animals in field cages in both terrestrial and aquatic systems could be amenable to our R_index_ approach.

Calculating R_index_, rather than using more conventional methods of assessing competitive ability such as a response surface design, could be useful for broad assessment of competitive ability for multiple species within a community. Not only do these methods enable us to test RCT for species ill-suited to flow-through systems like chemostats, but they also facilitate simultaneous assessment of competitive abilities of a greater number of species using the same resource. For example, to compare competitive abilities of a set of 5 species, a conventional response surface approach would require 10 pairwise competition experiments, with 10 different density and intraspecific/interspecific ratios per pairwise experiment. With our R_index_ approach, it may be possible to obtain competitive rankings for 5 species sharing a resource in only 5 single-species experiments with perhaps as few as 3 resource treatments per experiment. For assemblages of >5 species the disparity in experiment number becomes even greater.

It should be noted that the R_index_ approach, like R* in RCT, is limited to cases in which resource competition is the primary form of competition. Because of this, response surface designs will continue to be valuable for assessing interspecific competitive ability, particularly when the specific type of competition is unknown. R_index_ also does not assess indirect and nonadditive effects of competition that can occur within multispecies assemblages. Nevertheless, our R_index_ approach provides an alternative experimental tool for rapid assessment of resource competitive ability (as opposed to other mechanisms of competition), and its role in community dynamics. Possible examples of situations where this approach could be advantageous might include assessment of resource competitive ability of an invasive species relative to many natives, determination of the role of resource competition in metacommunities (such as in patch dynamics), and comparisons of resource competitive abilities of multiple species involved in ecological succession.

We have demonstrated the utility of nonequilibrium approaches to application of resource competition theory to metazoans in microcosm experiments. In the case of our system, the typical microcosm experiments, rearing cohorts for one generation with detritus resources input as a single pulse, results in a nonequilibrium experimental system that is probably representative of how these species interact in nature. In nature, containers receive pulses of detritus inputs, and then may go long periods with little input [28], [45], and in such situations, cohorts of mosquitoes may establish, grow, emerge, and then die out as resources are depleted. However, our approach is not dependent on this match of the nonequilibrium experiment with nonequilibrium natural situation. Even for systems that may reach equilibrium in nature (e.g., plants exploiting soil resources) short term microcosm experiments are common (e.g. [46]) and are best thought of as nonequilibrium experiments for which our approach should yield useful evaluations of competitive ability. We conclude that this general approach may be implemented in a variety of nonequilibrium systems, for a variety of organisms. Efforts to apply our R_index_ approach to other systems could help to further our understanding of the role of resource competition in a wide array of ecological communities.
