# Correction: Competitive Abilities in Experimental Microcosms Are Accurately Predicted by a Demographic Index for R*

**DOI:** 10.1371/annotation/2a92cb58-c4bf-4381-81ed-7770e01b5a78

**Published:** 2013-10-04

**Authors:** Ebony G. Murrell, Steven A. Juliano

There is an error in affiliation for author Ebony G. Murrell. She is not affiliated with #1 but with #2 School of Biological Sciences, Illinois State University, Normal, Illinois, United States of America. 

